# Enhanced Efficacy of Doxorubicin by microRNA-499-Mediated Improvement of Tumor Blood Flow

**DOI:** 10.3390/jcm5010010

**Published:** 2016-01-19

**Authors:** Ayaka Okamoto, Tomohiro Asai, Sho Ryu, Hidenori Ando, Noriyuki Maeda, Takehisa Dewa, Naoto Oku

**Affiliations:** 1 Department of Medical Biochemistry, University of Shizuoka School of Pharmaceutical Sciences, 52-1 Yada, Suruga-ku, Shizuoka 422-8526, Japan; s14803@u-shizuoka-ken.ac.jp (A.O.); asai@u-shizuoka-ken.ac.jp (T.A.); rorange0805@gmail.com (S.R.); h.ando@tokushima-u.ac.jp (H.A.); 2 Nippon Fine Chemical Co. Ltd., 5-1-1 Umei, Takasago, Hyogo 676-0074, Japan; n-maeda@nipponseika.com; 3 Department of Life and Materials Engineering, Nagoya Institute of Technology, Gokiso-cho, Showa-ku, Nagoya 466-8555, Japan; takedewa@nitech.ac.jp

**Keywords:** microRNA, miR-499, cancer therapy, gene delivery, liposomes, tumor blood perfusion

## Abstract

Genetic therapy using microRNA-499 (miR-499) was combined with chemotherapy for the advanced treatment of cancer. Our previous study showed that miR-499 suppressed tumor growth through the inhibition of vascular endothelial growth factor (VEGF) production and subsequent angiogenesis. In the present study, we focused on blood flow in tumors treated with miR499, since some angiogenic vessels are known to lack blood flow. Tetraethylenepentamine-based polycation liposomes (TEPA-PCL) were prepared and modified with Ala-Pro-Arg-Pro-Gly peptide (APRPG) for targeted delivery of miR-499 (APRPG-miR-499) to angiogenic vessels and tumor cells. The tumor blood flow was significantly improved, so-called normalized, after systemic administration of APRPG-miR-499 to Colon 26 NL-17 carcinoma–bearing mice. In addition, the accumulation of doxorubicin (DOX) in the tumors was increased by pre-treatment with APRPG-miR-499. Moreover, the combination therapy of APRPG-miR-499 and DOX resulted in significant suppression of the tumors. Taken together, our present data indicate that miR-499 delivered with APRPG-modified-TEPA-PCL normalized tumor vessels, resulting in enhancement of intratumoral accumulation of DOX. Our findings suggest that APRPG-miR-499 may be a therapeutic, or a combination therapeutic, candidate for cancer treatment.

## 1. Introduction

**Scheme 1:**
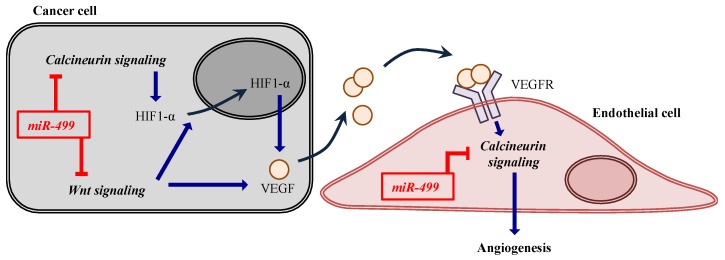
Associations of signaling pathways and miR-499. The blue arrows indicate upregulation. The red symbols mean inhibition.

miR-499 is one of the miRNAs that regulate the expression of several genes, especially under hypoxia/ischemia conditions such as those found in cancer, myocardial infarction, and so on [1]. It is known that miR-499 is involved in particular signaling pathways including Wnt signaling and calcineurin pathways [1,2]. It has also been reported that the Wnt signaling pathway is involved in carcinogenesis and cell differentiation, and the calcineurin pathway in cancer cell growth. Additionally, both of these signaling pathways induce angiogenesis [3,4]. It is well established that the calcineurin catalytic subunit α isoform (CnAα) is involved in the promotion of angiogenesis following activation of the transcriptional factor nuclear factor of activated T cells (NFAT). It is known that hypoxia-inducible factor 1-α (HIF1-α) is regulated by the calcineurin-NFAT pathway. HIF1-α causes vascular endothelial growth factor (VEGF) secretion from cells, resulting in promotion of angiogenesis. In addition, in some kinds of tumors, the Wnt signaling pathway also regulates the production of VEGF. Since miR-499 inhibits the Wnt and calcineurin signaling pathways through the suppression of target genes that construct these pathways, it has been expected that miR-499 might be a good candidate for cancer treatment (Scheme 1). Indeed, we showed earlier that miR-499 suppresses the growth of tumor cells and the capillary tube formation of human umbilical vein endothelial cells (HUVECs) *in vitro* [5].

A miRNA delivery system is necessary for application of miRNAs to cancer therapy. Previously, we developed a small RNA delivery system using tetraethylenepentamine-based polycation liposomes (TEPA-PCL) [6,7,8] and demonstrated that miR-499/TEPA-PCL reduces the secretion of VEGF and the production of other pro-angiogenic factors, e.g., CnAα and frizzled family receptor 8 (FZD8), in Colon 26 NL-17 mouse carcinoma cells [5]. For systemic administration and tumor targeting, we modified the Ala-Pro-Arg-Pro-Gly (APRPG) peptide on the surface of miR-499/TEPA-PCL (APRPG-miR-499) and showed that APRPG-miR-499 significantly suppresses tumor growth through silencing of target genes in Colon 26 NL-17-carcinoma–grafted mice [5]. Since it is shown that miR-499 could be a potential predictive biomarker in patients with lung cancer treated with chemotherapy [9], elucidation of miR-499 activity *in vivo* is of considerable interest.

In general, tumors construct angiogenic vessels to secure a supply route from blood to obtain oxygen and nutrients [10,11]. Since the production of tumor vessels is rapid, these vessels are immature and leaky, and are chaotically constructed, resulting in loops or dead-end conformations. These factors lead to intratumoral hypertension, which causes difficulty in delivery of anti-cancer drugs into the tumors [12]. Since these vessels do not extend to all parts of the tumors, some regions in the tumor tissue become severely hypoxic. Fewer blood vessels mean not only poor delivery of anti-cancer drugs, but also a hypoxic environment. Such an environment works negatively when the anti-cancer drug has been administered as follows: Tumors metabolize the nutrients anaerobically in hypoxia, resulting in the accumulation of lactic acid in the tumor tissue, which causes acidosis. In this case, the efficacy of basic anti-cancer drugs such as doxorubicin and paclitaxel is decreased dramatically. Therefore, the normalization of tumor blood vessels is a meaningful goal in cancer therapy. Inhibition of angiogenesis ameliorates blood perfusion in the tumor due to the decrease of defective tumor vessels without blood flow. Anti-cancer drugs that extend deeply into the tumors via improved blood flow are expected to show potent cytotoxic effects. Recent studies showed that the combination therapy of anti-angiogenic agents and anti-cancer drugs is effective for the treatment of cancer [13,14,15,16,17]. Such therapy is based on the concept of vascular normalization, *i.e.*, the tumor vessels temporally show the function similarly to normal vessels [18,19,20]. In fact, Avastin^®^, an anti-VEGF antibody, which has been used as an anti-angiogenic agent in combination therapies with anti-cancer drugs, causes the extension of progression-free survival in the clinical setting [21].

In the present study, we focused on the anti-angiogenic effect of miR-499. We developed APRPG-miR-499 as a partner of DOX for combination therapy and examined its therapeutic effects in tumor-grafted mice.

## 2. Experimental Section

### 2.1. Preparation of Tetraethylenepentamine-Based Polycation Liposomes (TEPA-PCL) and Lipoplex

Cholesterol-conjugated miR-499 (miR-499-C) and cholesterol-conjugated control miRNA (miCont-C) were purchased from Hokkaido System Science (Hokkaido, Japan). The 3′ end of the passenger strand of miRNAs was modified with cholesterol as previously reported [7,8]. miR-499 was composed of miR-499-5p and miR-499-3p. The sequences of the strands were as follows: miR-499-5p: 5′-UUAAGACUUGCAGUGAUGUUU-3′, miR-499-3p: 5′-AACAUCACAGCAAGUCUGUGCU-3′. Dicetyl phosphate-tetraethylenepentamine (DCP-TEPA) was synthesized as described earlier [6]. Cholesterol, dipalmitoylphosphatidylcholine (DPPC), dioleoylphosphatidylethanolamine (DOPE), and Ala-Pro-Arg-Pro-Gly (APRPG)-grafted polyethylene glycol (6000)-distearoylphosphatidylethanolamine (APRPG-PEG-DSPE) were synthesized by Nippon Fine Chemical (Hyogo, Japan).

TEPA-PCL was prepared as described previously [6]. DOPE, cholesterol, DPPC, and DCP-TEPA (4:4:3:1 as a molar ratio) were mixed and lyophilized overnight. Then the lipid was hydrated with diethylpyrocarbonate (DEPC)-treated water. These liposomes were extruded 10 times through a polycarbonate membrane filter with a pore size of 100 nm (Nuclepore, Maidstone, UK). To form miR-499-C/TEPA-PCL lipoplexes, we mixed miR-499-C with TEPA-PCL in DEPC-treated water at a ratio of the nitrogen moiety derived from TEPA-PCL to the phosphorus from miR-499-C (N/P ratio) of 18 and incubated this mixture for 20 min at room temperature. In order to apply them to systemic administration, miR-499-C/TEPA-PCL were modified with APRPG-PEG-DSPE. APRPG-PEG-DSPE at a ratio of 10% of total lipids was incubated with miR-499-C/TEPA-PCL at 50 °C for 20 min to obtain APRPG-modified miR-499-C/TEPA-PCL lipoplex (APRPG-miR-499). Cholesterol-conjugated non-targeting miRNA (miCont-C) instead of miR-499-C was used to prepare APRPG-modified miCont-C/TEPA-PCL lipoplex (APRPG-miCont). The particle size and ζ-potential of APRPG-miR-499 diluted with 10 mM phosphate buffer were measured with a Zetasizer Nano ZS (Malvern, Worcestershire, UK).

### 2.2. Cell Culture

Colon 26 NL-17 mouse carcinoma cells were established by Yamori (Japanese Foundation for Cancer Research, Tokyo, Japan) and were kindly provided by Nakajima (SBI Pharmaceuticals, Tokyo, Japan). The cells were cultured in DMEM/Ham’s F-12 medium (Wako, Osaka, Japan), which was supplemented with 10% fetal bovine serum (FBS, AusGeneX, Oxenford, Australia), 100-units/mL penicillin G (MP Biomedicals, Irvine, CA, USA), and 100-µg/mL streptomycin (MP Biomedicals), in a CO_2_ incubator.

### 2.3. Experimental Animals

BALB/c mice (male, five weeks old) were purchased from Japan SLC (Shizuoka, Japan). The animals were cared for according to the Animal Facility Guidelines of the University of Shizuoka. All animal experiments were approved by the Animal and Ethics Committee of the University of Shizuoka on september 18, 2013 (Approved No. 136048).

For preparation of tumor-bearing mice, Colon 26 NL-17 cells (1 × 10^6^ cells/mouse) were implanted subcutaneously into the left posterior flank of BALB/c mice. Lipoplex samples were administered via a tail vein at selected times after the implantation, as described in each experiment.

### 2.4. Evaluation of Tumor Blood Flow

APRPG-miR-499 or APRPG-miCont (2 mg/kg as miRNA) lipoplexes were intravenously injected into the tumor-bearing mice seven days after the implantation. The mice were injected Biotynylated Lycopersicon esculentum (tomato) Lectin (Vector laboratories, Inc., Burlingame, CA, USA) four days after administration of APRPG-miRNAs in order to stain the vessels with blood perfusion. Ten minutes later, the mice were fixed by reflux flow with 1% paraformaldehyde. The tumors were excised and embedded in Tissue-Tek^®^ O.C.T. Compound (Sakura Finetek Japan, Tokyo, Japan). Frozen tumor sections of 10 µm thickness were prepared with a Microm HM 505 E Cryostat (Micro-edge Instruments, Tokyo, Japan) and mounted on MAS-coated slides (Matsunami Glass, Osaka, Japan). After having been fixed with acetone and blocked with 3% bovine serum albumin in phosphate-buffered saline (PBS), these tumor sections were incubated with streptavidin-Alexa Fluor^®^ 594 conjugate (Life Technologies, Gaithersburg, MD, USA) for 1 h in a humid chamber, and then with anti-mouse CD31 (PECAM-1) antibody labeled with FITC (eBioscience, San Diego, CA, USA) for 1 h. The fluorescence of Alexa Fluor^®^ 594-labeled Lycopersicon esculentum Lectin and FITC-labeled CD31 was observed by confocal laser-scanning microscopy (LSM 510 META, Carl Zeiss, Oberkochen, Germany). To obtain the percentage of vessels with blood flow, we divided the co-localized area of Alexa Fluor^®^ 594 (blood flow area) and FITC (total vessels) by the FITC-positive area (total vessels).

### 2.5. Accumulation of Doxorubicin in the Tumor

APRPG-miR-499 or APRPG-miCont (2 mg/kg as miRNA) lipoplexes were intravenously injected into the tumor-bearing mice seven days after the implantation. Four days later, the mice were intravenously injected with DOX at a dose of 10 mg/kg. The tumors were refluxed with PBS and excised 3 h after the injection. After the tumors had been homogenized in 0.3 M HCl in 70% ethanol by use of a ShakeMan 2 (Biomedical Science, Tokyo, Japan), the fluorescence of DOX (excitation wavelength (Ex.): 470 nm, emission wavelength (Em.): 590 nm) were measured with Infinite M200 (TECAN, Männedorf, Switzerland).

### 2.6. Therapeutic Experiment

For investigation of APRPG-miR-499 mono-therapy, tumor-bearing mice were prepared and injected with APRPG-miR-499 (2 mg/kg as miR-499) intravenously on the day when the tumor size had reached 50 mm^3^. The tumor size and body weight of each mouse were monitored daily from day four after the injection. Tumor volume was calculated from the following formula: 0.4 × a × b^2^ (a; largest diameter, b; smallest diameter). On the other hand, for the combination therapy, the tumor-bearing mice were administered APRPG-miR-499 or APRPG-miCont (2 mg/kg as miRNAs) intravenously as described above. Then, at four days after the injection, the mice were injected intravenously with DOX at a dose of 5 mg/kg. The tumor size and body weight were monitored as mentioned above.

### 2.7. Statistical Analysis

The statistical differences in more than three groups were evaluated by analysis of variance (ANOVA) with the Tukey *post-hoc* test, whereas those in two groups were determined by using Student’s *t* test.

## 3. Results and Discussion

### 3.1. Amelioration of Incomplete Blood Flow in Tumors Treated with miR-499

**Figure 1:**
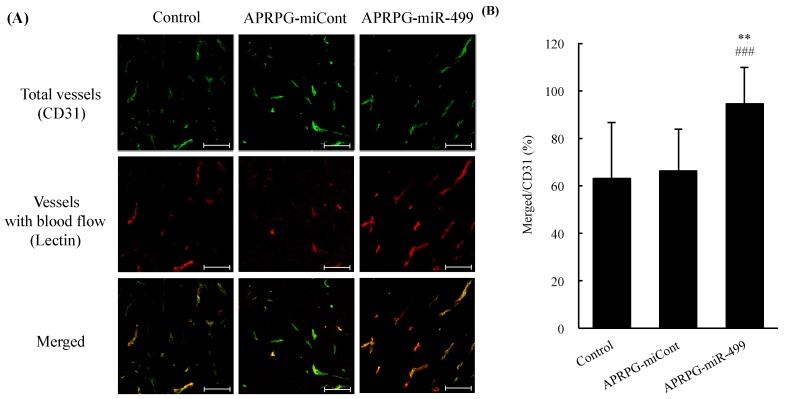
miR-499-mediated improvement of blood flow in tumor blood vessels. Colon 26 NL-17 cells were subcutaneously implanted into the left posterior flank of BALB/c mice. APRPG-miR-499 or APRPG-miCont (2 mg/kg as miRNA) were administered intravenously seven days after the implantation. Perfused vessels were labeled by intravenous injection of biotin-conjugated Lycopersicon esculentum Lectin at 96 h after the lipoplex injection. The tumor vessels were fixed by reflux flow with 1% paraformaldehyde. After the solid tumors had been dissected, 10-μm frozen sections were prepared. CD31 of the vasculature was immunostained with FITC. Biotin-conjugated Lycopersicon esculentum Lectin was labeled with Streptavidin-Alexa Fluor^®^ 594. Green indicates vasculature, red indicates vessels with blood flow. Yellow color indicates areas of double-stained vessels. (**A**) Confocal images are shown. Scale bars indicate 100 µm; (**B**) Percent lectin^+^CD31^+^ double-positive area/total CD31^+^ area was determined to assess perfusion efficiency of the tumor vasculature. Data are presented as percent ratio of merged area/CD31^+^ area. Asterisks indicate significant differences (** *p* < 0.01 *vs.* control, ^###^ *p* < 0.001 *vs.* APRPG-miCont).

Physicochemical properties of APRPG-miR-499 were as follows: particle size, 163 ± 13 nm; ζ-potential, −0.30 ± 0.46 mV. Because the complexes of miR-499 and TEPA-PCL (lipoplexes) themselves had a strong positive charge (*ca.* +45 mV), we modified the surface of the lipoplexes with PEG6000 in order to mask the charge and prolong their blood circulation. Our previous study revealed that APRPG-modified TEPA-PCL accumulates in tumor tissues after intravenous injection and becomes associated with tumor endothelial cells owing to the potent affinity of APRPG for VEGF receptor 1 (VEGFR 1) on the endothelial cells [5]. Therefore, it would be expected that APRPG-modified TEPA-PCL would enable miR-499 to arrive at the tumor tissues and to become internalized effectively in the target cells. By confocal microscopic observation at four days after the administration of miR-499 to mice, we found that the tumor vessels had much more blood flow compared with the APRPG-miCont–injected group (Figure 1A). Quantitative analysis showed that the tumor vessels that had been treated with APRPG-miR-499 had blood flow about 1.5 times higher than that of the other groups, although the number of blood vessels was not significantly different between APRPG-miR-499 and APRPG-miCont (Figure 1B). The proportion of vessels with blood perfusion was about 60% in the APRPG-miCont–treated group; however, more than 90% of the vessels had blood flow after the APRPG-miR-499 treatment, indicating that miR-499 might have affected the improvement of tumor blood vessels. Previously we reported that miR-499 inhibits the capillary tube networks *in vitro* [5]. We consider that miR-499 regulated the balance of pro-angiogenic factors and anti-angiogenic ones by inhibiting VEGF secretion.

### 3.2. Improvement of Doxorubicin (DOX) Accumulation in Tumors Treated with miR-499

**Figure 2:**
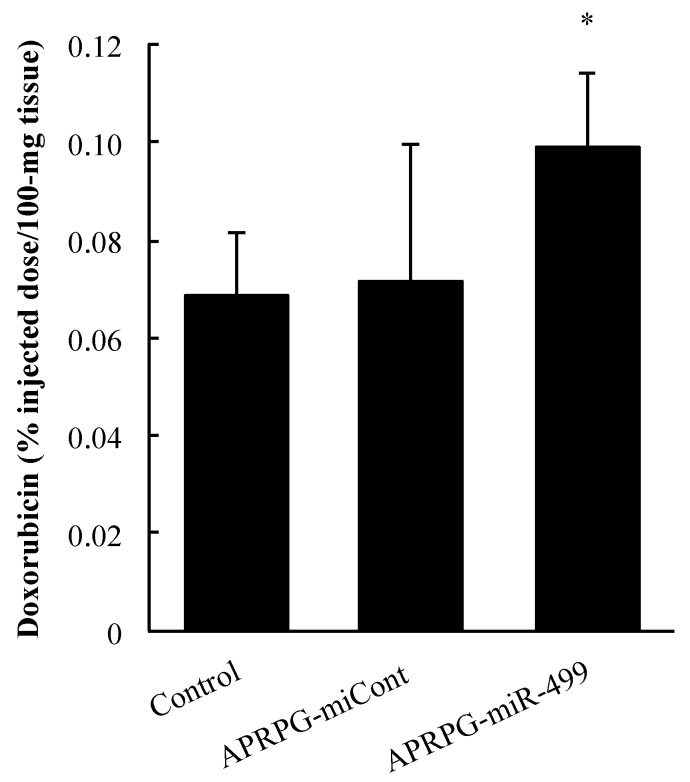
Improvement of DOX accumulation in tumors treated with miR-499. Colon 26 NL-17 cells were subcutaneously implanted into the left posterior flank of BALB/c mice. APRPG-miR-499 or APRPG-miCont were administered intravenously seven days after implantation. Four days after the lipoplex injection, DOX was administered intravenously. Three hours after DOX injection, tumor tissues were excised. The tumors were homogenized with ShakeMan2 and then centrifuged. DOX accumulation was quantified by measuring the fluorescence of DOX (Ex. 470 nm, Em. 590 nm). Asterisks indicate significant differences (* *p* < 0.05).

The accumulation of DOX in the tumors four days after its administration to the tumor-bearing mice was determined by measuring the fluorescence of DOX. The results showed that more DOX accumulated in the tumor tissue in the miR-499-treated mice than in the other groups (Figure 2). Since the tumor blood vessels treated with miR-499 had the blood perfusion as mentioned, a high amount of DOX was carried to the tumor tissue with blood perfusion. In general, it is known that tumor vessels are defective and leaky because of rapid angiogenesis [12]. Additionally, lymphatic dysfunction in tumors causes intratumoral hypertension, resulting in less accumulation of an anti-cancer drug in the deeper part of the tumor. The inhibition of VEGF production results in the regression of incomplete tumor blood vessels and drives the improvement of the vessels [13,14,15]. The normalization of tumor vessels leads to reduced stromal pressure, which increases the amount of anti-cancer drugs delivered to the tumors [22,23]. Our findings suggest that miR-499 could enhance the accumulation of DOX in the tumors due to the development of a favorable environment for drug delivery via VEGF regulation.

### 3.3. Combination Therapy with miR-499 and DOX

**Figure 3:**
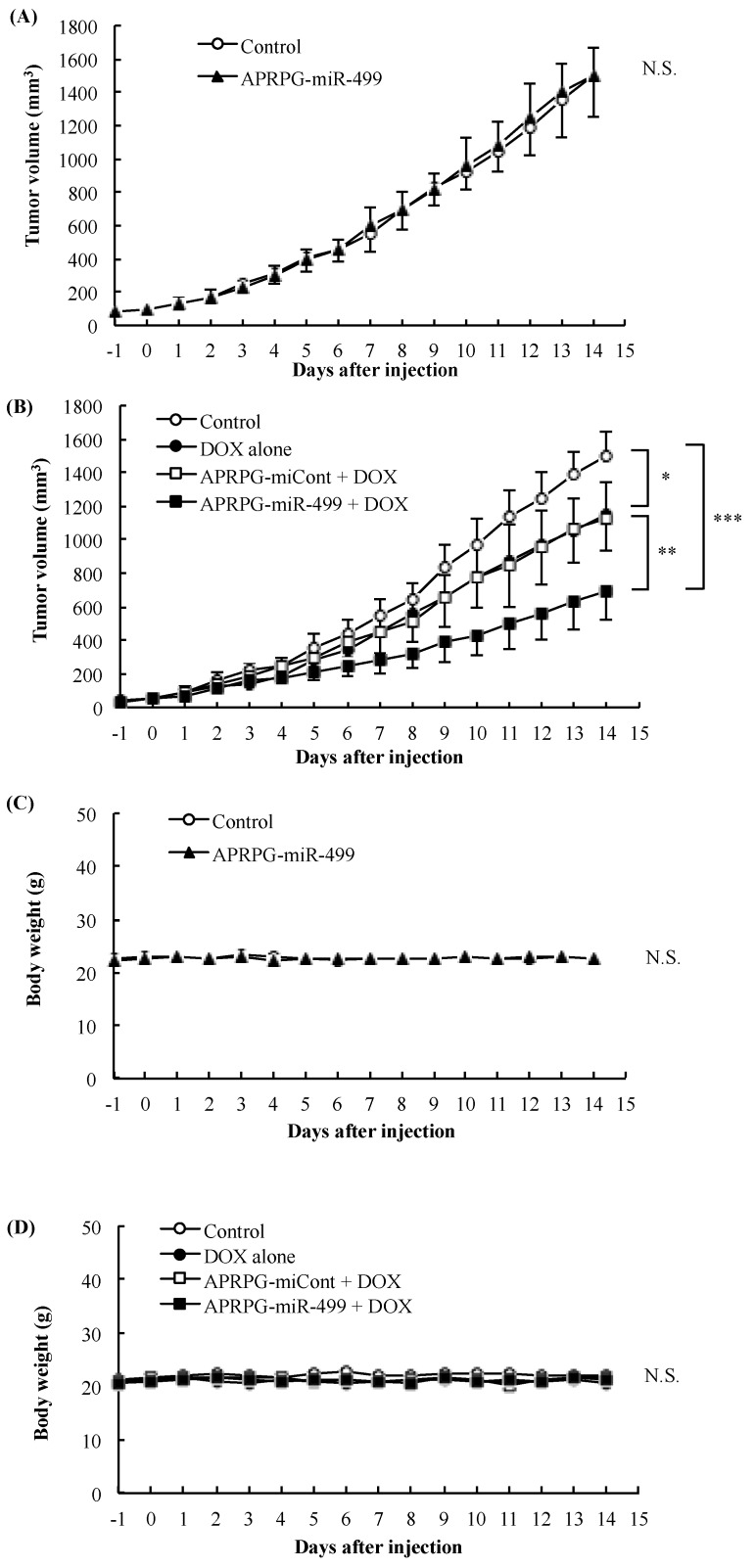
Combination therapy with miR-499 and DOX. Colon 26 NL-17 cells were subcutaneously implanted into the left posterior flank of BALB/c mice. APRPG-miR-499 was administered intravenously when the tumor volume had reached 50 mm^3^. In the case of combination therapy, DOX (5 mg/kg) was intravenously injected via a tail vein at four days after the lipoplex injection. The tumor size (**A**,**B**); and body weight (**C**,**D**) of each mouse were monitored daily from one day before lipoplex injection. Asterisks indicate significant differences (* *p* < 0.05, ** *p* < 0.01, *** *p* < 0.001). N.S. means no significant difference.

For the therapeutic experiment, tumor-bearing mice were intravenously administered APRPG-miR-499 and/or DOX. Although APRPG-miR-499 did not inhibit the tumor growth with a single injection at a dose of 2 mg/kg as miR-499 (Figure 3A), it did enhance the antitumor effect of DOX (Figure 3B). These data suggest that APRPG-miR-499 contributed to the normalization of blood flow in the tumor vessels, allowing DOX to accumulate in the tumor tissue and resulting in an enhanced antitumor effect of DOX. Previously, we reported that APRPG-miR-499 monotherapy caused a significant inhibition of tumor growth when given twice to mice bearing smaller tumors at a dose of 2 mg/kg/day as miR-499 [5]. These data indicate that a low dose of miR-499 mainly improved blood flow due to the normalization of incomplete tumor blood vessels, and a high dose mainly reduced blood vessels themselves due to the suppression of angiogenesis, resulting in the inhibition of tumor growth. Also, importantly, there was no weight loss in any group after the injection (Figure 3C,D). Considering the clinical application of anti-cancer drugs, it is meaningful that a smaller amount of DOX would be enough for the treatment of cancer. We expect that combination therapy of miR-499 and DOX would enable patients to have reduced side effects and to maintain their quality of life at a high level. These results suggest that miR-499 could be a good candidate for increasing the efficacy of anti-cancer drugs. Taken together, our data indicate that miR-499 enhanced the effect of an anti-cancer drug, DOX, possibly through vascular normalization, suggesting that combination therapy with miR-499 and anti-cancer drugs can be a potential therapeutic strategy.

## 4. Conclusions

In the present study, we found that the tumor blood flow was increased significantly after the administration of APRPG-miR-499. In addition, the accumulation of DOX in the tumor tissue was also increased after the treatment with APRPG-miR-499. Furthermore, the tumor growth was suppressed significantly with DOX and miR-499 combination therapy compared with DOX monotherapy. Our findings provide a novel strategy for cancer therapy and contribute to the development of genetic drugs and/or therapeutic modalities based on RNA interference.
